# Salinomycin induces autophagic cell death in salinomycin-sensitive melanoma cells through inhibition of autophagic flux

**DOI:** 10.1038/s41598-020-75598-1

**Published:** 2020-10-28

**Authors:** Yajing Liu, Yinghua Hao, Yuxia Li, Yadan Zheng, Jiajing Dai, Fubo Zhong, Wei Wei, Zhengyu Fang

**Affiliations:** 1 Biomedical Research Institute, Shenzhen Peking University-Hong Kong University of Science and Technology Medical Center, Shenzhen, 518036 China; 2 Department of Thyroid and Breast Surgery, Peking University Shenzhen Hospital, Shenzhen, 518036 China; 3 Clinical Research Institute, Shenzhen Peking University-Hong Kong University of Science and Technology Medical Center, Shenzhen, 518036 China; 4 Department of Medical Genetics, Institute of Basic Medical Sciences, Chinese Academy of Medical Sciences, Beijing, 100005 China

**Keywords:** Chemotherapy, Melanoma, Macroautophagy

## Abstract

Several literature has shown that salinomycin (Sal) is able to kill various types of cancer cells through different signaling pathways. However, its effect on melanoma has seldom been reported. We examined the anti-cancer efficacy of Sal in melanoma cell lines, and found six of eight cell lines were sensitive to Sal. Given the fact that the roles of Sal are diverse in different cancer types, we were eager to figure out the mechanism involved in the current study. We noticed the most sensitive line, SK-Mel-19, showed a typical morphological change after Sal treatment. The autophagy inhibitor, 3-MA, could effectively suppress Sal-induced cell death. It could also facilitate the increase of autophagic markers and reduce the turnover of autophagosomes, which resulted in an aberrant autophagic flux. On the other hand, Sal could stimulate endoplasmic reticulum stress and cause an accumulation of dysfunctional mitochondria. We also discovered a potential correlation between *LC3B* mRNA level and its sensitivity to Sal in 43 clinical melanoma samples. Overall, our results indicated that Sal could have multiple effect on melanoma cells and induce autophagic cell death in certain kinds of cells, which provided a new insight into the chemotherapy for melanoma.

## Introduction

Melanoma, which is transformed from melanocytes, is becoming the most aggressive form of skin cancer worldwide^1^. Incidence and natural history of melanoma is affected by age and racial background with ultraviolet radiation (UVR) often as the main etiological factor^2^. The behavior of melanoma can be affected by melanogensis, which is an essential process of melanocytic cells^3^. In recent years, advances in chemo-, radio- and immunotherapies have been made in the treatment of melanoma, yet these successes are restricted by the swift development of drug resistance^4–6^. So the discovery of novel classes of drugs and additional therapeutic options are desperately needed to combat this malignant tumor.

Salinomycin (Sal), a potassium ionophore^7^, is a monocarboxylic acid polyether antibiotic which is previously used as an antibacterial drug and antioxidant^8,9^ (Supplementary Fig. S1). Regardless of its toxicity, it is potentially the first promising compound screened through high-throughput techniques based on cancer stem cell theory. Sal selectively targets breast cancer stem cells in vitro and inhibits breast tumor dissemination, growth and metastasis in vivo^10^. Despite the well-known anticancer effect of Sal, exact mechanism is not clear^11^. Several reports have addressed questions of the modality of cell death induced by Sal, but there is still no consensus. Most studies support that Sal triggers cell apoptosis, an essential mechanism by which anticancer drugs damage tumor cells^12–14^, while others have proposed necrosis and autophagic cell death^15–17^.

Macroautophagy (autophagy) has recently emerged as an essential regulator of cell death pathways involved in cancer initiation, development, and progression^18–21^. Autophagy is a highly conserved lysosomal degradation pathway that contributes to cellular homeostasis by degrading damaged or redundant organelles, and misfolded proteins^22–24^. In response to stressful conditions, it provides nutrients and energy to cells by reducing endoplasmic reticulum (ER) stress and limiting the production of reactive oxygen species (ROS)^25^. Thus, it is quite conceivable that abnormal autophagy can eventually destroy cells. The term “Autophagic cell death” is employed to define the instance in which the autophagy machinery is required for cell death^26,27^. However, so far, autophagic cell death is primarily a morphologic definition (i.e. cell death associated with autophagosomes/autolysosomes), and there is still no conclusive evidence that a specific mechanism of autophagic death actually exists^28^.

In the present study, we exhibited that Sal could suppress the fusion between autophagosomes and lysosomes and induce autophagic cell death in Sal-sensitive melanoma cells. Furthermore, we showed a potential sensitive marker LC3B for Sal. These are novel findings in melanoma, and may help in designing more effective and less toxic therapeutic strategies for its treatment.

## Results

### Salinomycin showed diverse killing effect on different melanoma cell lines

**Figure 1:**
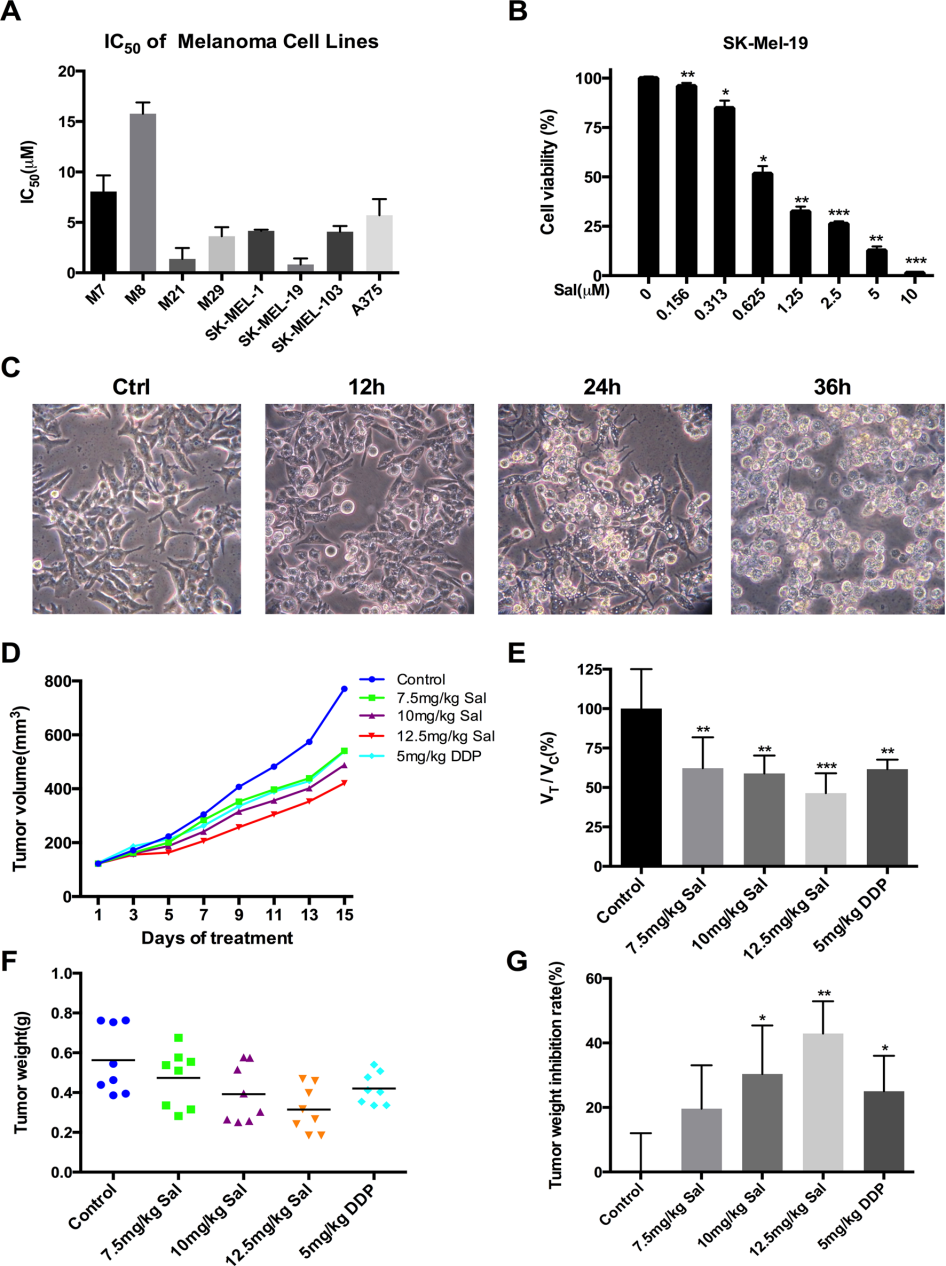
Anti-cancer effect of salinomycin in melanoma cell lines. (**a**) IC_50_ of eight melanoma cell lines were detected by MTS assay after treated with Sal at the indicated concentrations (0–40 μM) and calculated by GraphPad Prism. (**b**) SK-Mel-19 cells were treated with different concentrations of Sal for 72 h, and cell viability was tested by MTS assay. (**c**) Changes in the morphology of SK-Mel-19 cells. Cells after treatment with 1.0 μM Sal for the indicated period of time were examined by phase-contrast microscopy (magnification, × 200). (**d**) Tumor volume growth curve of five groups. (**e**) Comparison of the relative tumor proliferation rate V_Treatment_/V_Control_ of five groups (n = 8). (**f**) The excised tumors were weighed at the end point. The scatter diagram of tumor weight was shown. (**g**) Tumor weight inhibition rate of five groups were calculated (n = 8). (*p < 0.05; **p < 0.01; ***p < 0.001 vs control).

As shown in Fig. 1a, the sensitivities of melanoma cell lines (M7, M8, M21, M29, SK-MEL-1, SK-MEL-19, SK-MEL-103 and A375) to Sal were examined. Most melanoma lines, except for M7 and M8, were sensitive to Sal. Among them, SK-Mel-19 (IC_50_ = 0.82 ± 0.60 μM) was extremely susceptive where 5 μM Sal could induce about 90% cell death in 24 h (Fig. 1b). The morphology change of SK-Mel-19 cells after Sal treatment was dramatic. Visible vacuoles started to accumulate in the cytoplasm of SK-MEL-19 cells upon 12 h treatment, which became more and larger afterwards. Most of the cells detached from the surface and burst in 36 h (Fig. 1c).

We further examined the effect of Sal on SK-Mel-19 in vivo. Treatment of Sal (7.5 mg/kg) resulted in a remarkable inhibition of tumor growth compared to normal control, whose effect was similar to 5 mg/kg cisplatin (DDP) (Fig. 1d). The relative tumor proliferation rate V_Treatment_/V_Control_ in the Sal groups was slower than that of the control group (Fig. 1e). The body weight of mice showed a significant decrease in the DDP group while varied little in the Sal group due to the serious side effect of DDP (Supplementary Fig. S2A). Two weeks after first treatment, mice were killed and tumor tissues were dissected, photographed and assessed (Supplementary Fig. S2B). The inhibition ratio of tumor weight indicated that Sal had a dose-dependent inhibitory effect on the tumor growth in vivo (Fig. 1f,g).

### Salinomycin-induced cell apoptosis is not the main cause of cell death

**Figure 2:**
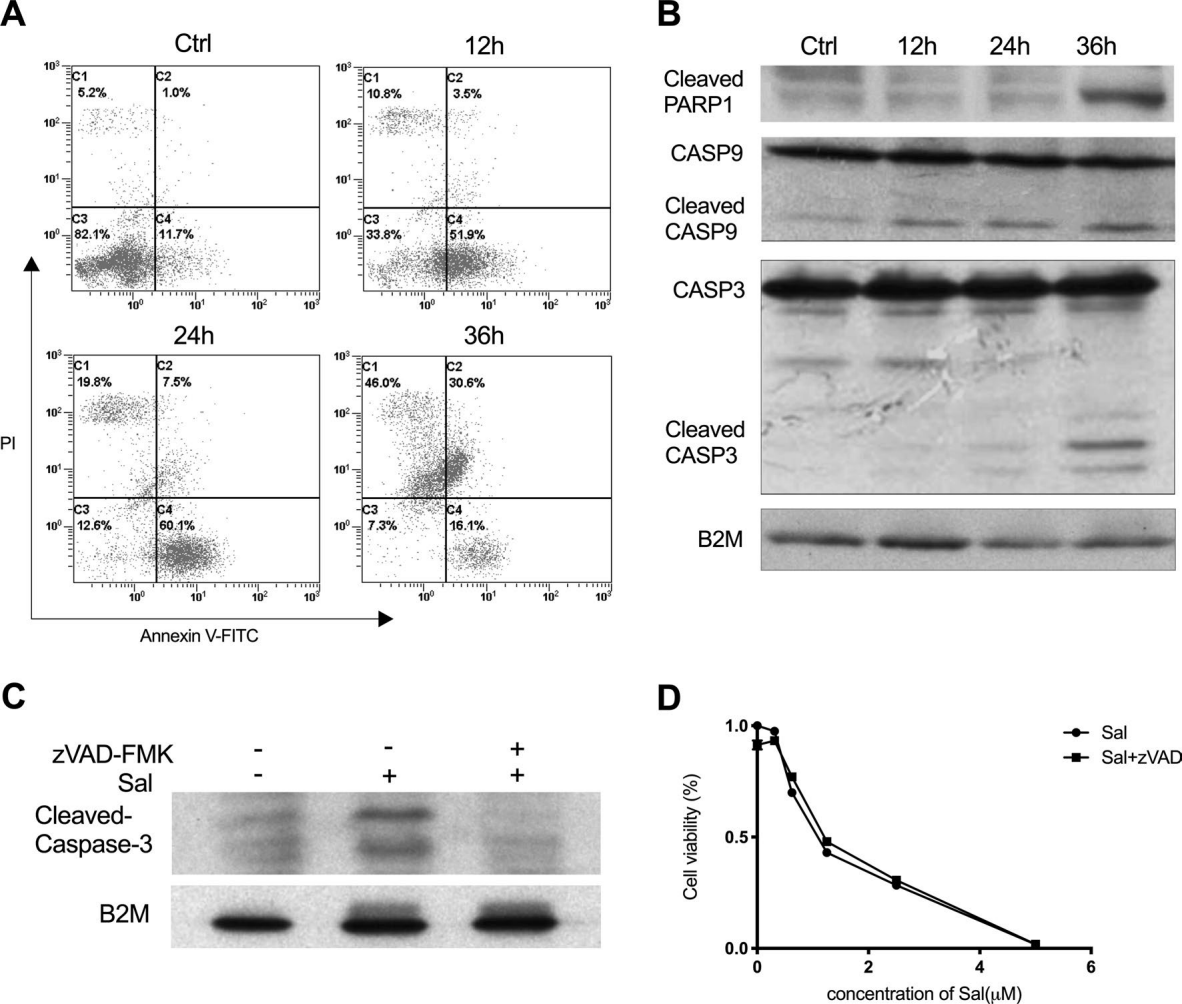
Apoptosis induced by salinomycin in SK-Mel-19 cells. (**a**) Cells treated with 1.0 μM Sal were stained with Annexin V-FITC/PI and detected by flow cytometry analysis. (**b**) Levels of protein expression were measured by western blot analysis using antibodies against PARP1, Caspase9, and Caspase3. (**c**) Samples treated with Sal alone (1.0 μM, 24 h), or with Sal (1.0 μM) and zVAD-FMK (20 μM) for 24 h immunoblotted for cleaved-Caspase3. (**d**) Cell viability of SK-Mel-19 cells treated with different concentrations of Sal alone for 24 h or Sal combination with zVAD-FMK (20 μM) for 24 h was measured by MTS assay.

To investigate the cause of cell death induced by Sal in SK-Mel-19, we conducted the Annexin V-FITC and PI staining to detect the apoptotic index. After 12–24 h treatment of Sal, the number of early apoptotic cells was elevated compared to the control. The apoptotic cells at later stage increased significantly at 36 h (Fig. 2a). We measured the cleavages of PARP1, Caspase3 and Caspase9 at different time points of treatment. There was a detectable increase of the cleavaged PARP1 and Caspase3 in cells treated with Sal for 36 h, while there was no change before 24 h. The cleavage of Caspase9 could be detected earlier (12 h) and increased along with the time of Sal treatment (Fig. 2b). These results suggested Sal might induce caspase-dependent apoptosis^29^. We thus treated cells with broad-spectrum caspase inhibitor zVAD-FMK and assessed the cell viability induced by the combination of Sal and zVAD-FMK or single reagent. As shown in Fig. 2c, zVAD-FMK could reduce the cleavage of Caspase3 evidently. However, there was no difference of cell viability between cells treated with and without zVAD-FMK along with Sal treatment (Fig. 2d). Furthermore, typical apoptosis morphological feature was only observed in a few SK-Mel-19 cells under transmission electron microscope, such as chromatin condensation, DNA fragmentation and chromatin margination (Supplementary Fig. S3). These findings indicated that apoptosis might not be the main cause of Sal-induced cell death.

### Increase of autophagic markers in the Sal-treated SK-Mel-19 cells

**Figure 3:**
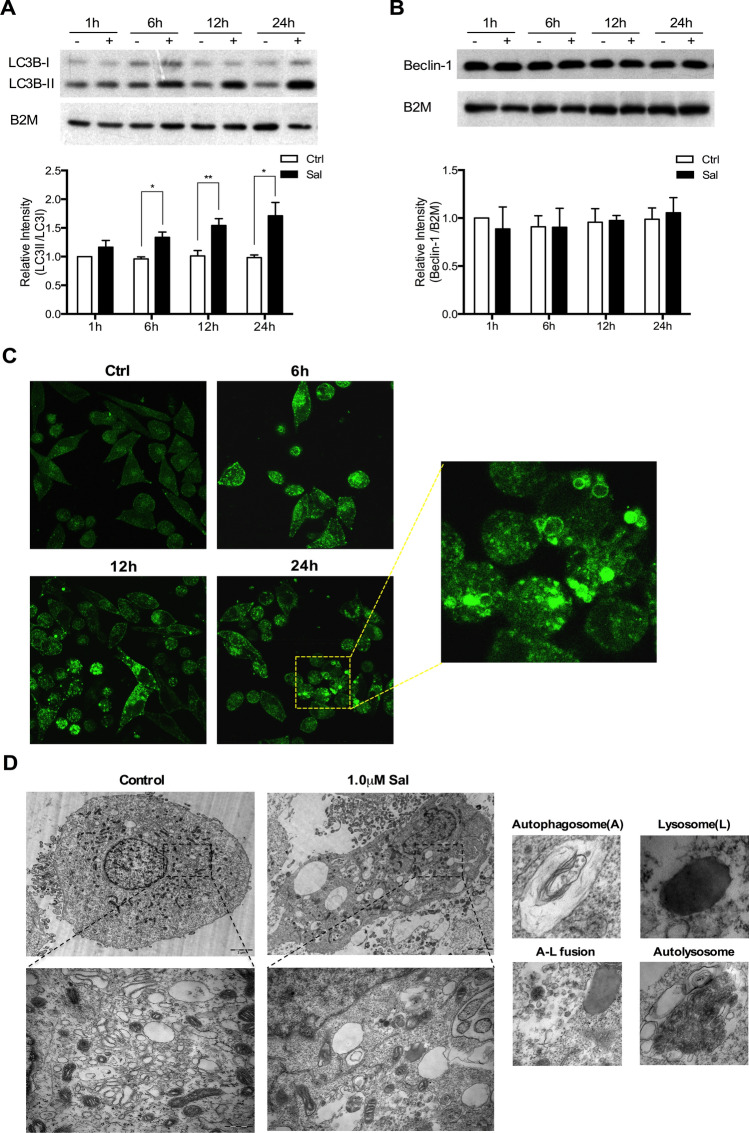
Increase of autophagic markers in Sal-treated cells. (**a**) Samples from DMSO-treated control and Sal-treated (1.0 μM) cells immunoblotted for LC3B (*p < 0.05; **p < 0.01; n = 3). (**b**) Samples from DMSO-treated control and Sal-treated (1.0 μM) cells immunoblotted for Beclin-1 (n = 3). (**c**) SK-MEL-19-GFP-LC3B cells were treated with 1.0 μM Sal for the indicated period. (**d**) TEM images of SK-Mel-19 cells treated with or without 1.0 μM Sal for 24 h. Individual portraits of autophagosomes and lysosomes were represented in the subpanel along with the images showing autophagosome, lysosome fusion and autophagolysosome.

In the microscopic observation, we noticed that treatment of Sal on SK-Mel-19 cells resulted in the vacuolization of cell contents, which might be associated with cell autophagy. Thus, we examined the expression of LC3B protein, a widely used autophagic marker, in SK-Mel-19 cells treated with or without Sal (1 μM). We found that LC3B-II significantly increased upon Sal treatment in a time dependent manner, which indicated the accumulation of lipidation of LC3B-I and formation of LC3B-II (Fig. 3a), while the protein level of Beclin-1, another protein relevant to autophagy^30^, remained unchanged (Fig. 3b).

To monitor autophagosome formation, we constructed a SK-MEL-19 cell line stably expressing the GFP-LC3B fusion gene (SK-MEL-19-GFP-LC3B). Most of GFP-LC3B proteins were diffusely distributed in the cytoplasm of control cells without treatment. However, there was a time-dependent increase of perinuclear GFP-LC3B-positive aggregates after Sal treatment, indicating the formation of autophagosomes (Fig. 3c). We performed transmission electron microscopy (TEM), one of the most valid and convincing methods, to detect autophagic compartments. Sal induced strong vacuolization and multiple autophagosomes formed in SK-Mel-19 cells. Individual portraits of phagosomes and lysosomes were represented along with the images showing autophagosome, lysosome formation and mature autolysosome (Fig. 3d).

### Autophagic flux is limited in SK-Mel-19 cells after salinomycin treatment

**Figure 4:**
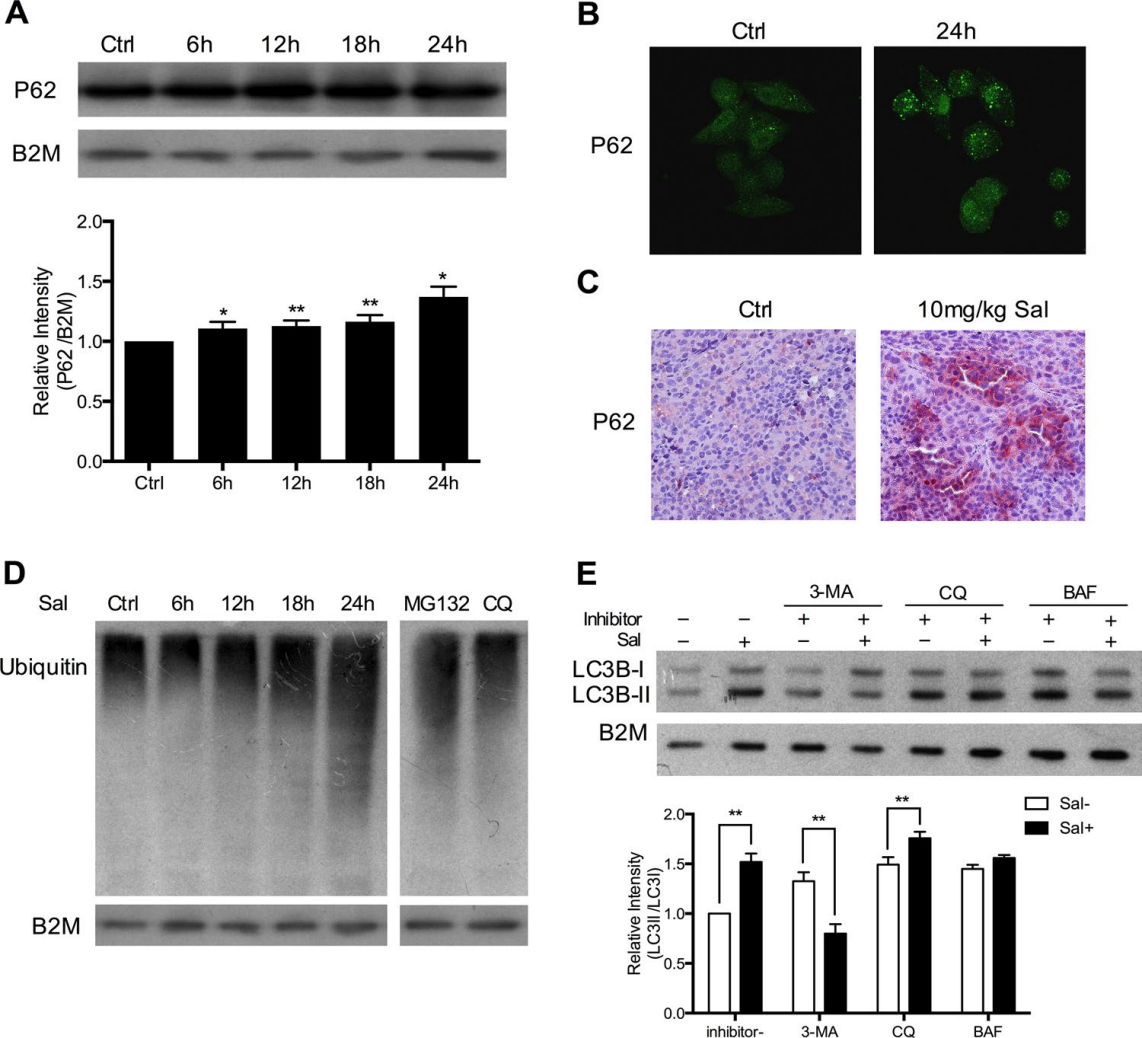
Reduction of autophagic flux in Sal-treated SK-Mel-19 cells. (**a**) Samples from DMSO-treated control and Sal-treated (1.0 μM) cells immunoblotted for P62 (*p < 0.05; **p < 0.01; n = 3). (**b**) Immunofluorescent assay showed the expression of P62 in SK-Mel-19 cells treated with or without 1.0 μM Sal for 24 h. (**c**) The expression level of P62 was examined by immunohistochemical analysis in 10 mg/kg Sal-treated SK-Mel-19 tumor xenograft. (**d**) Samples treated with 1.0 μM Sal for the indicated period of time or MG132 (5 μM) for 6 h or CQ (6.25 μM) for 6 h immunoblotted for ubiquitinated proteins. (**e**) SK-Mel-19 cells was treated with Sal alone, inhibitor alone, or their combination for 24 h. Level of protein expression of LC3B was measured by western blot analysis (**p < 0.01; n = 3).

Autophagy is a complex and dynamic process that consists of several sequential steps-sequestration, transport to lysosomes, degradation, and utilization of products degradation^31^. An accumulation of autophagosomes can be attributed to either an increase of autophagic activity or a reduced turnover of autophagosomes, which can be indicated by the amount of degraded proteins^27^. We wondered whether there was activation of protein degradation by autophagy in Sal-treated cells. P62 protein that serves as a link between LC3 and ubiquitinated substrates becomes incorporated into completed autophagosomes and degrades in autolysosomes^32,33^. Therefore, the increased level of P62 indicates the inhibition of autophagic degradation. As shown in Fig. 4a P62 levels increased in a time-dependent manner after cells treated with Sal (1.0 μM). To validate the change, immunofluorescence (IF) and immunohistochemistry (IHC) analysis were performed. P62 protein increased and gathered in the cytoplasm after Sal treatment (Fig. 4b,c). Recent evidence also indicates that the inhibition of autophagy causes an increase of ubiquitinated proteins^34^. We found that ubiquitinated proteins accumulated gradually after treatment (Fig. 4d). MG132 (an inhibitor of proteasomes) and CQ (an inhibitor of autophagy) here were used as positive controls. The results indicated that there was an inhibition of autophagic degradation in Sal-treated cells.

Inhibitors of autophagy can function at different stages of autophagic process^27^. Here we chose three classic autophagic inhibitors: 3-MA (PI3-kinase inhibitor), CQ (Lysosomal lumenalkalizer) and BAF (Vacuolar-type H^+^-ATPase inhibitor) that respectively acted at sequestration steps or post-sequestration steps for further studies. We examined the co-effect of these inhibitors with Sal. As shown in Fig. 4e, LC3B-II was obviously decreased after Sal-treated in combination with 3-MA compared to Sal alone, which indicated the impairment of autophagosome formation. On the other hand, when cells were treated with Sal in combination with CQ or BAF, LC3B-I was slightly increased. These not only indicated the presence of autophagic flux inhibition, but also provided evidence that Sal mainly acted on post-sequestration steps after autophagosomes had formed. Thus, Sal might have a similar mechanism to CQ.

### Failure of the fusion between autophagosomes and lysosomes results in autophagic cell death upon Sal treatment

**Figure 5:**
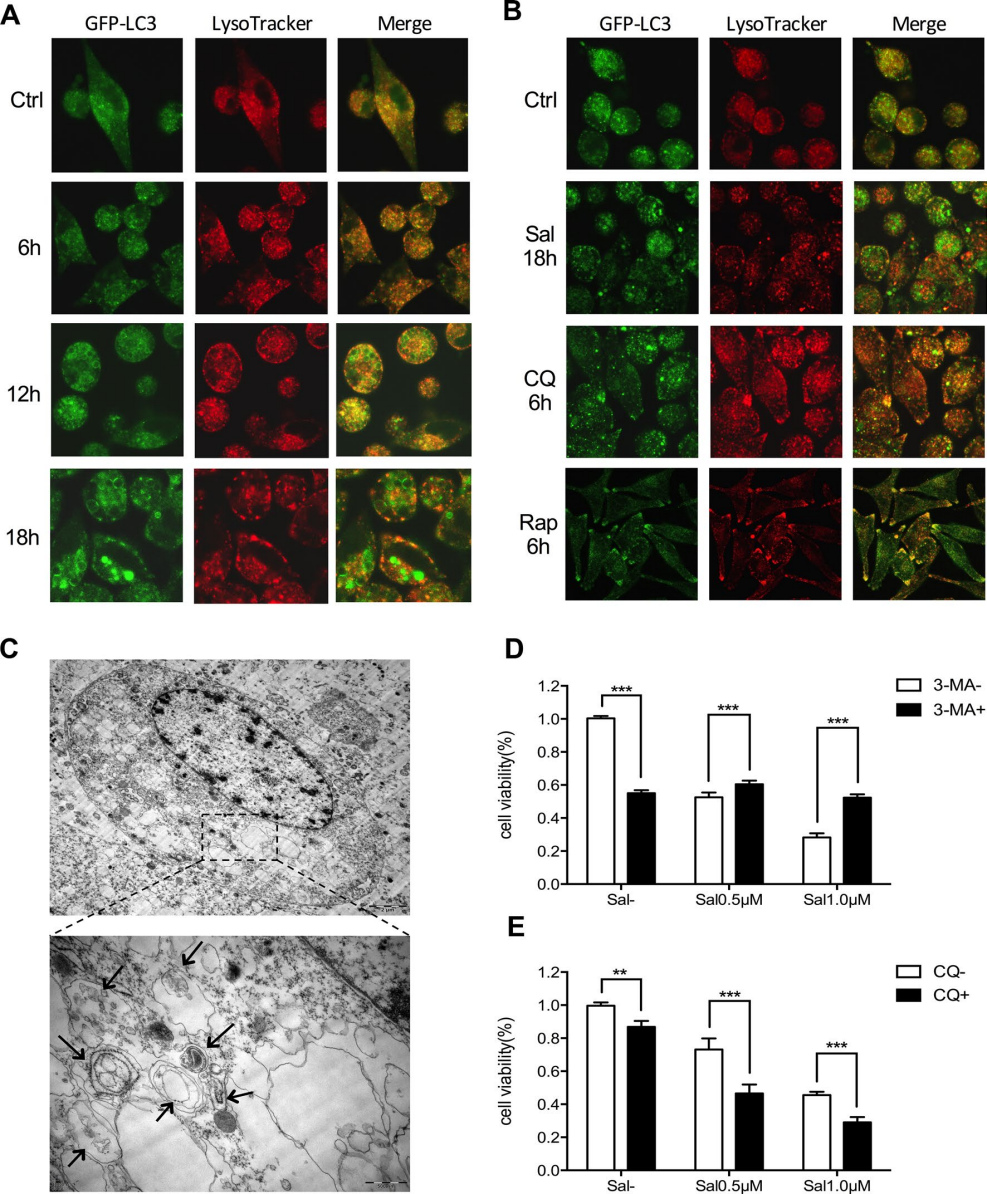
Failure of the fusion between autophagosomes and lysosomes. (**a**) Live-cell confocal microscopic images of SK-Mel-19-GFP-LC3B cells stained with LysoTracker Red treated with 1.0 μM Sal for 6 h or 12 h or 18 h (magnification, × 630). (**b**) Live-cell confocal microscopic images of SK-Mel-19-GFP-LC3B cells stained with LysoTracker Red treated with 1.0 μM Sal for 18 h, or treated with CQ for 6 h, or Rap for 6 h (magnification, × 630). (**c**) TEM images showing many autophagosomes, large-scale autophagic vacuolization of the cytoplasm and resultant vacuolated appearance in SK-Mel-19 cells after treated with Sal (1.0 μM) for 36 h.[Scale bars, 2 μm (Above) and 500 nm (Below).] (**d**, **e**) Bars denoted cell viability of SK-Mel-19 cells treated with Sal (0.5 μM, 1.0 μM) alone or combination with 3-MA (2.5 mM) or CQ (6.25 μM) respectively (**p < 0.01; ***p < 0.001).

Then we wondered whether the fusion of autophagosomes with lysosomes, the final stage of autophagy, was influenced by Sal treatment. We stained SK-Mel-19-GFP-LC3B cells with LysoTracker Red (an acidic pH marker for lysosomes) and performed imaging using live-cell confocal microscopy. As shown in Fig. 5a, adequate signal separation was observed in cells treated with 1 μM Sal, indicating that the fusion between autophagosomes and lysosomes was severely impaired. Then we used Rap (an autophagy inducer) and CQ as negative and positive controls. After cells treated with Rap, the signals of LC3B and lysosomes were significantly overlapped (Fig. 5b). Interestingly, we found lysosomes were greatly dilated in CQ-treated cells but not altered (still small and complete) in Sal-treated cells. Thus, Sal could prevent the autophagosome-lysosome fusion in SK-Mel-19 cells, while the mechanism within this progress was different from that of CQ.

Given the significantly altered autophagic flux in SK-Mel-19 cells upon Sal treatment, we next examined whether this could lead to autophagic cell death. Until now, autophagic cell death was still mainly a morphologic definition. We did observe the obvious change of morphology in SK-Mel-19 cells upon Sal treatment. Moreover, TEM imaging showed the existence of autophagosomes, large-scale autophagic vacuolization of the cytoplasm and resultant vacuolated structures in the cells treated with Sal (Fig. 5c). More importantly, we also observed that SK-Mel-19 cells treated with Sal showed the phenotype of cell swelling, nucleus and organelle degeneration and loss of membrane integrity. Although the definition of autophagic cell death is still under disagreement, the most widely accepted criterion is the inhibition of cell death by various autophagy inhibitors^35^. Using this criterion, the results remained controversial because 3-MA succeeded to improve cell viability after combined with Sal, while CQ failed to reduce cell death caused by Sal. Instead, CQ seemed to have synergistic effect with Sal (Fig. 5d,e). Considering that Sal was an inhibitor of autophagy in SK-Mel-19 cells, the mechanism described here was a different type of autophagic cell death compared with the regular type, which were associated with increased autophagosomes and reduced degradative capability.

Similar approaches were performed in M21, another Sal-sensitive cell line, and similar results were obtained (data not shown here), indicating the therapeutic effect of Sal on certain kinds of melanoma.

### Salinomycin also induces ER stress and mitochondrial dysfunction in SK-Mel-19 cells

**Figure 6:**
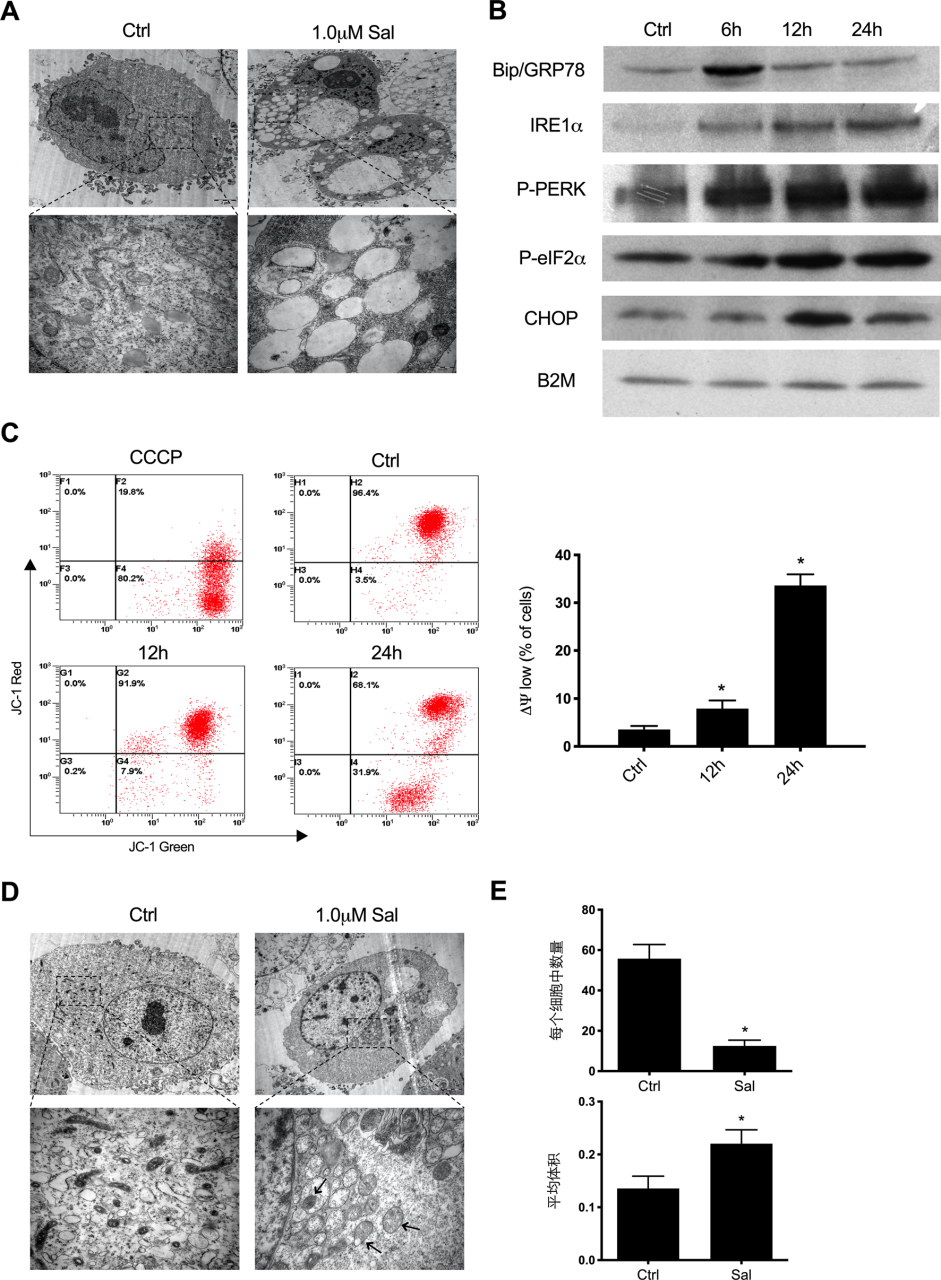
Induction of ER stress and mitochondrial dysfunction in SK-Mel-19 cells. (**a**) EM images showing dilated ER and accumulation of smooth ER in 1.0 μM Sal-treated SK-Mel-19 cells.[Scale bars, 2 μm (Above) and 500 nm (Below).] (**b**) Samples treated with 1.0 μM Sal for the indicated period of time immunoblotted for ER stress related proteins. (**c**) Changes in JC-1 green and red fluorescence in SK-Mel-19 cells treated with Sal for 12 h or 24 h were detected by flow cytometer. The percentage of cells with low ΔΨ_m_ was determined (*p < 0.05; n = 3). (**d**) EM images showing abnormally looking mitochondria with fewer and swollen cristae in 1.0 μM Sal-treated SK-Mel-19 cells. [Scale bars, 2 μm (Above) and 500 nm (Below).] (**e**) The number of functional mitochondria and average size of each mitochondrion were quantified, n = 5–6 micrographs per condition (*p < 0.05).

It has been reported that Sal can up-regulate and activate several key endoplasmic reticulum (ER) stress proteins^36^, which might be correlated with the anti-tumor effect of Sal. Here we also examined whether ER stress was influenced by Sal treatment in SK-Mel-19 cells. As shown in Fig. 6a, the results of TEM revealed that ER was extensively distended and dilated in cells with Sal treatment and that the associated ribosomes on ER were nearly completely lost. These results indicated the presence of ER stress.

ER stress can activate a complex signaling network, the unfolded protein response (UPR), to restore homeostasis^37^. Here we also examined the alteration of several ER stress-associated proteins that might be influenced by Sal treatment. The expression of Bip/GRP78 increased significantly at 6 h and returned to baseline at 12 h and 24 h after Sal treated, while the expression of IRE1α gradually increased with time. These proved the appearance of ER stress and activation of UPR. We also found that the phosphorylation of PERK and eIF2α was enhanced by Sal treatment, which could decrease the initiation of translation. The expression of CHOP, the downstream effector of PERK-eIF2α-ATF4 pathway, was also increased (Fig. 6b).

ER stress can promote mitochondrial damage. Autophagy is responsible for removing and cycling damaged mitochondria^38^. Meanwhile, studies have shown that Sal can alter ΔΨ_m_ of cells^39^. We found that SK-Mel-19 cells treated with Sal displayed lower ΔΨ_m_ than the cells without Sal treatment, indicating the possibility of alteration in mitochondrial functionality (Fig. 6c). Through TEM analysis we observed that the abnormal mitochondria were shorter in the cells treated with Sal compared to the controls, which contained fewer and swollen cristae (Fig. 6d). Moreover, the number of mitochondria with intact morphological features was reduced, while there was an increase of mitochondria size. These demonstrated that the function of mitochondria had been badly influenced (Fig. 6e). Therefore, we assumed that ER stress and mitochondrial damage maybe the cause of the increase of autophagosomes.

### *LC3B* mRNA level might be a sensitive marker for Sal in melanoma

**Figure 7:**
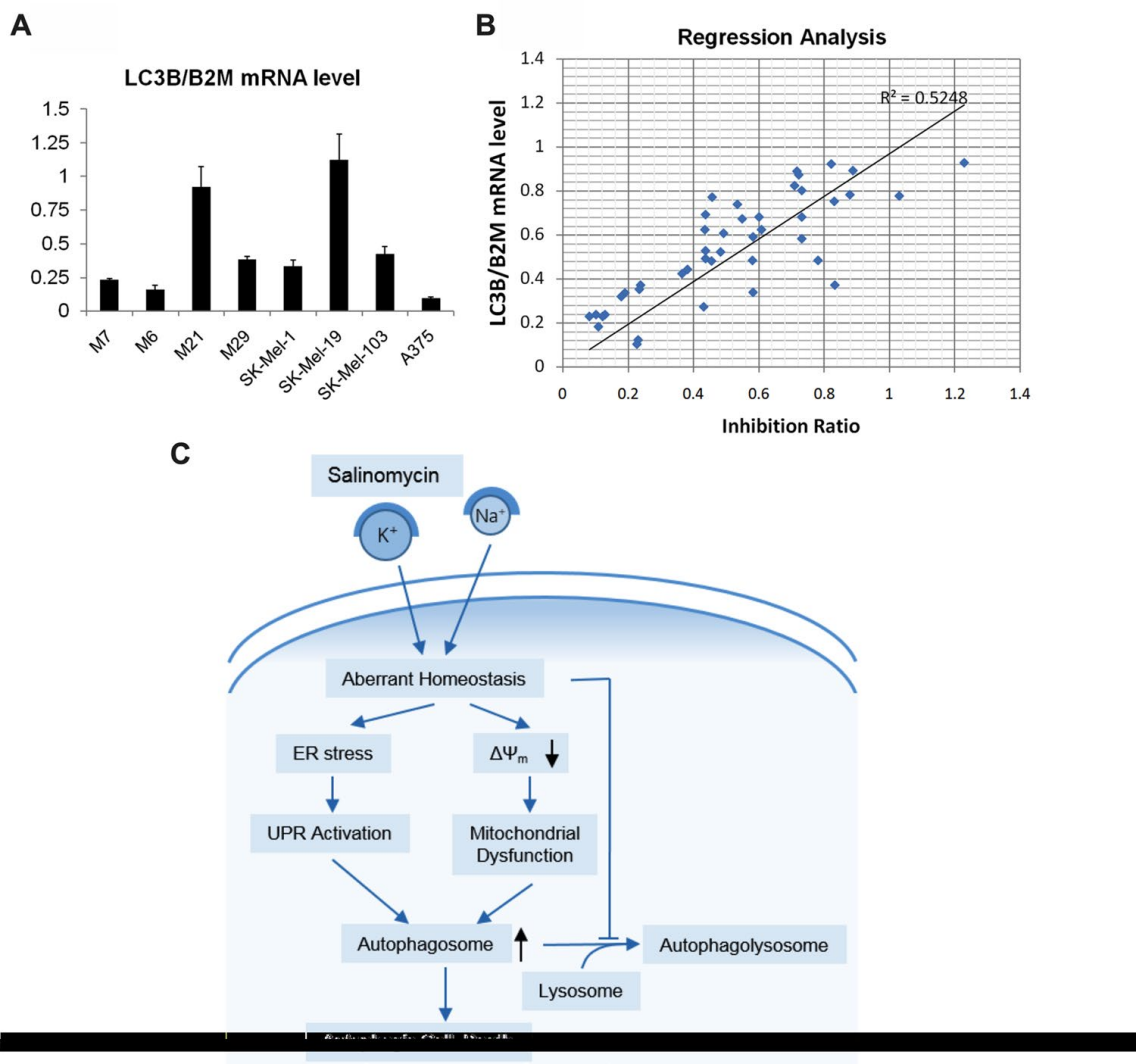
A correlation between *LC3B* mRNA level and sensitivity of Sal in melanoma. (**a**) The mRNA levels of *LC3B *were analyzed using real-time PCR assay, and normalized with the amount of *B2M* mRNA. (**b**) Linear regression analysis was performed to identify the potential correlation between *LC3B* expression and inhibition rate of Sal treatment (1 μM, 7 days) on clinical melanoma samples. (**c**) Model of the action of Sal in SK-Mel-19 cells. Sal which disrupted the balance of Na^+^ and K^+^ triggered ER stress and a decrease of mitochondrial ΔΨ, thus leading to UPR and damaged mitochondria. To keep its stability balance and normal function, cells produced autophagosomes to degrade those damaged organelles. However, Sal not only induced autophagy, but also inhibited the fusion between autophagosomes and lysosomes, causing an aberrant autophagic flux. Finally, accumulations of autophagosomes lead to autophagic cell death.

When we examined the expression of autophagy-related genes in melanoma lines, we occasionally found that *LC3B* mRNA was especially highly expressed in the Sal-sensitive lines (M21 and SK-Mel-19, Fig. 7a). We wondered whether there was a correlation between *LC3B* mRNA expression and sensitivity of Sal in melanomas. We used the *in-vitro* drug sensitivity approach to identify the sensitivity of Sal in 43 clinical melanoma samples and performed the regression analysis. As shown in Fig. 7b, there was a positive correlation between *LC3B* mRNA level and inhibition ratio induced by Sal treatment in clinical melanoma samples.

## Discussion

Although there have been some new therapies for the treatment of melanoma occurred in recent years, cancer cells have become gradually resistant to drugs and the time needed for this process is shorter and shorter. Therefore, the need to find new drugs, which have different mechanisms of action, is extremely urgent. Salinomycin, a famous anticancer drug, has multiple pathways to inhibit tumor growth and has effect on various kinds of cancer cells. However, there is still no research of Sal on melanoma.

Here we figured out that Sal had cell-killing effect on melanoma, especially on SK-Mel-19 cell line. Meanwhile, some cell lines that were sensitive to Sal were not sensitive to the clinical commonly used drug cisplatin, including M29, SK-Mel-19, and A375 cell line (Supplementary Table S2). Some studies have indicated that Sal can specially damage tumor cells that are insensitive to drugs induced apoptosis, including drug resistant cancer cells^40,41^. Our study provided a new case to this field and showed that the mechanism of Sal was not limited to apoptosis.

Some articles have reported that Sal can induce autophagy in some kinds of cells, which plays a pro-survival role and attenuates the apoptotic cascade^36,39^. While others have found that Sal induces an aberrant autophagic flux, which are associated with the production of apoptosis^13,14^. In the present study, we discovered that Sal could induce autophagy in Sal-sensitive melanoma cell lines (M21, M29, SK-Mel-1, SK-Mel-19, SK-Mel-103 and A375 cell line). Among them, the change of autophagic flux in SK-Mel-19 cells was extremely obvious, which might be the reason of its sensitivity. In SK-Mel-19 cells, we observed that autophagic flux was blocked at post-sequestration steps because of the failure of fusion between autophagosomes and lysosomes. Latest study has proposed that the inhibition effect of Sal might act on the potential of lysosomal membrane^16^. However, some researchers indicated that Sal inhibited lysosomal activity instead of the autophagosome and lysosome fusion^14^.

Rossitza et al. showed that some autophagosomes contained melanized melanosomes, accounting for the phenomenon of “coarse melanin” in malignant melanoma^42^. The findings suggested that autophagy could be a constitutive metabolic state for invasive and metastatic melanoma cells. In the current study, a “coarse melanin” was also observed using TEM when Sk-Mel-19 cells were treated by Sal (Fig. 3d). Considering that melanoma cells are characterized by melanogenesis and pigmentation, more details of the effect of Sal on melanoma need to be verified. Melanogenesis shortens overall- and disease free-survival of patients with stage III and IV disease. The presence and type of melanin pigment (eumelanin vs. pheomelanin) affects melanoma development in animal models^43^. Some research has also found that melanogenesis regulates the melanoma behavior by affecting HIF-1-dependent pathways and can affect survival in patients with stage III and IV melanoma^44–46^. Moreover, pigmentation of melanoma cells affects its sensitivity to some drugs/compounds, which indicates that pigmentation plays an important role in therapeutic resistance^47,48^. By examining the melanin level, it`s a pity that there is no evident correlation between the sensitivity to Sal and the pigmentation of eight melanoma cell lines (Supplementary Fig. S4). However, the change of melanogenesis and pigmentation after Sal treated may directly relate to Sal clinical application, which is worth more research.

Several findings have demonstrated that Sal increases ROS levels, suggesting that high levels of ROS are an overall consequence of Sal treatment^13,15,17^. Here we found that Sal could induce expansion of endoplasmic reticulum (ER) and the reduction of ribosomes around ER. Western blot results indicated ER stress was caused by Sal, thus caused the UPR response. At the same time, the number of disabled mitochondria increased, suggesting that there was mitochondrial dysfunction. The changes of ER and mitochondria could lead to intracellular autophagy occurred to maintain cell homeostasis. In the meantime, autophagic flux was inhibited by Sal. Even autophagosome membrane had been produced to encapsulate these dysfunctional organelles, they could not be degraded, causing the accumulation of autophagosomes to promote cell death (Fig. 7c). In our study, we also found that the source of autophagosome membrane was not only from endoplasmic reticulum, but also from mitochondria, for the mitochondrial membrane gradually expanded and wrapped in some substances such as mitochondria or endoplasmic reticulum.

On the other hand, ferroptosis appears with the production of ROS. Thus, Sal might also cause ferroptosis through induction of ROS. However, ferroptosis is characterized morphologically by the presence of smaller mitochondria with condensed mitochondrial membrane densities. Here in the cells treated by Sal, we observed the abnormal mitochondria had bigger average size and contained fewer and swollen cristae. There is no direct evidence of the presence of ferroptosis.

There is a lot of controversy over the mechanism of cell death caused by Sal, which includes apoptosis, necrosis and autophagic cell death. Here we found changes of apoptosis related proteins in SK-Mel-19 cells, but caspase inhibitor cannot reverse cell death. Surprisingly, the autophagy inhibitor 3-MA could partially reverse cell death. Therefore, we speculate that Sal influences cells mainly through autophagic cell death, and partly through apoptosis. The autophagic cell death described here is distinct from the traditional understanding of autophagic cell death that excessive autophagy causes cytotoxicity. Here it is an over-inhibited autophagy that causes cell death, which has a characteristic of the increase of autophagosomes and large-scale autophagic vacuolization of the cytoplasm, without obvious change of nucleus and cell membrane. This is a kind of cell death by autophagy not with autophagy.

It was shown that malignant melanoma cells display high levels of autophagy, and LC3B could be a marker for the cells’ autophagic status^42^. In the current study, we also noticed cell lines with high *LC3B* mRNA level were more sensitive to Sal, therefore we enrolled 43 patients to verify our assumption. We found the mRNA level of *LC3B* was positively correlated with sensitivity of Sal in local melanoma patients, which has the potential to be a sensitive marker for Sal treatment in melanoma. This was also a novel finding. However, more clinical trials with patients enrolled should be performed before it would be applied to clinical practice.

In summary, we propose that Sal induces a direct alteration of ER and mitochondria, inducing autophagy in SK-Mel-19 cells. While autophagic flux is inhibited by Sal at the same time, resulting in autophagic cell death. Meanwhile, LC3B can be a biomedical marker for Sal clinical use. Based on summing up other studies’ conclusions, Sal can cause different mechanisms of cell death, indicating that it has potential in treating a wide variety of cancers.

## Materials and methods

### Cell line and cell culture

Human melanoma cancer cell lines M7, M8, M21, M29, SK-MEL-1, SK-MEL-19, SK-MEL-103 and A375 were obtained from Jennio Biotech. (Jennio, China). All cells except SK-Mel-1 cells were cultured in Dulbecco’s Modified Eagle Medium (DMEM, Gibco, USA) supplemented with 10% fetal bovine serum (FBS, Gibco, USA), 100 IU/ml penicillin (Gibco, USA), and 100 μg/ml streptomycin (Gibco, USA). SK-Mel-1 cells were cultured in RPMI 1640 medium (Gibco, USA) supplemented with 10% FBS and antibiotics. All cell lines were grown in a 5% CO_2_ humidified incubator at 37 °C and maintained at a confluence of ~ 30%.

### Drugs and reagents

Salinomycin (Sal; Melonepharma, China), Rapamycin (Rap; Sigma-Aldrich, USA), Chloroquine (CQ; Sigma-Aldrich, USA), Bafilomycin (BAF; Selleck, USA), 3-Methyladenine (3-MA; Selleck, USA), benzyloxycarbonyl-Val-Ala-Asp-fluoro-methylketone (zVAD-FMK; Sigma-Aldrich, USA) and carbobenzoxyl-L-leucyl-L-leucyl-L-leucine (MG132; Sigma-Aldrich, USA) were used to treat cells. All drugs were dissolved in their respective buffers as per required concentration. Primary antibodies [LC3B (#3868), Beclin-1 (#4122), SQSTM1 (p62) (#7695), β2-microglobulin (B2M) (#12851), Caspase-3 (#9665), Caspase-9 (#9508), BiP (#3177), IRE1α (#3294), Phospho-PERK (#3179), CHOP (#5554), Phospho-eIF2α (#3398)] were obtained from Cell Signaling Technology (USA), whereas primary antibodies [Ubiquitin (ab134953), PARP (ab191217)] were purchased from Abcam (USA). The secondary antibodies mouse Anti-rabbit IgG (#5127) and rabbit Anti-mouse IgG (#58802) antibodies were purchased from Cell Signaling Technology (USA).

### Cell viability assay

Cells were plated in 96-well plates at a density of 3 × 10^3^ cells/well and treated with Sal and/or other drugs at different concentrations. After 72 h treatment, cell viability was measured by using MTS assay kit (Promega, USA) according to the manufacturer's protocol. The absorbance at 490 nm (A490) of each well was read on iMark Microplate Absorbance Reader (Bio-Rad, USA). Cell viability rates were represented as the percentage of corresponding control^15^. GraphPad Prism was used to draw the curve and calculate the half-maximal inhibitory concentration (IC_50_ value).

### In vivo study

The animal studies were authorized by the Animal Ethic Review Committees of Shenzhen Peking University-Hong Kong University of Science and Technology Medical Center. Forty female BALB/c nude mice, aged 4 weeks, were purchased from Beijing Vital River Laboratory Animal technology (Beijing, China) and were fed under standard pathogen-free conditions. SK-Mel-19 cells (0.5 × 10^6^) suspended in 100 µl PBS and 100 µl Matrigel (Corning, USA) were injected into the right axilla of each mouse. When tumor volume reached approximately 100 mm^3^, mice were randomized into five groups with eight mice in each group. Carboxymethylcellulose sodium (CMC-Na), Sal (7.5 mg/kg), Sal (10 mg/kg), Sal (12.5 mg/kg), and Cisplatin (DDP, 5 mg/kg) were used for the in vivo experiments. Sal (7.5, 10 and 12.5 mg/kg) group and control (CMC-Na only) group were given by gavage once a day for 14 days. Cisplatin injection was administered by intraperitoneal injection in DDP group (5 mg/kg). The length and width of the tumor were monitored by caliper measurement every 2 days and tumor volume was calculated according to the following formula: Volume = length × width^2^/2. At the end of the experiment, tumors were excised, photographed, and weighed. The tumor weight inhibition rate was calculated using the formula: Inhibition rate (%) = [(C − T)/C] × 100%, where C is the average tumor weight of the control group and T is the average tumor weight of the treated group^27^. All animal experiments were strictly implemented in compliance with the NIH Guide for the Care and Use of Laboratory Animals.

### Western blot analysis

After cells were treated with different drugs or different times, whole-cell lysates (20 μg/per lane) were separated by electrophoresis (Bio-rad, USA). Then proteins were transferred to PVDF membranes (Pall Corporation, Life Sciences). Membranes were incubated with primary antibody solution at 4 °C overnight after blocked, and then incubated with secondary antibody solution for an hour. Images were captured by densitometric scanning (Tanon-5500, Shanghai, China) and analyzed by ImageJ software^41^.

### Live-cell confocal microscopy

SK-MEL-19-GFP-LC3B cells were seeded onto 6 cm cell culture plates with 3 × 10^4^ per plate. After cell adherence, cells were stained with 75 nM solution in dimethyl sulfoxide (Lyso-Tracker Red; Beyotime, China) in DMEM for 2 h in a humidified CO_2_ incubator. Then cells were cultured in cell culture with different drugs for indicated time. Live-cell confocal images were photoed using LSM 510 META confocal microscope (Carl Zeiss, Germany).

### Transmission electron microscopy

Cells treated with Sal were fixed with 2.5% Glutaraldehyde for an hour at room temperature and for three hours at 4 °C. Then we used ethanol to gradually dehydrate the cells and Epon 812 to embed it. Ultrathin sections after specific treatment were photographed with JEOL JEM 1230 electron microscope at 100 kV.

### Measurement of mitochondrial membrane potential

After treatment with 1.0 μM Sal for 12 h or 24 h, cells were stained with 5,5′,6,6′-Tetrachloro-1,1′,3,3′-tetraethyl-imidacarbocyanine iodide (JC-1, Beyotime) at 37 °C for 20 min and then analyzed by Beckman flow cytometer (Beckman, China). The fluorescence ratio represented the change of mitochondrial membrane potential. Cells treated with carbonyl cyanide 3-chlorophenylhydrazone (CCCP, Beyotime) were used as positive control.

### Annexin V-FITC/PI binding assay

The Annexin V-FITC/PI flow cytometric assay kit (KeyGEN bioTECH, China) were used. Cells were incubated with binding buffer containing FITC-conjugated Annexin V and PI for 15 min at a density of 1.0 × 10^6^ cells/ml. We obtained and analyzed the scatter plots of FITC fluorescence versus PI fluorescence by Beckman flow cytometer (Beckman, China)^11^.

### RNA extraction and real-time PCR analysis

Total RNA was isolated from tissues by using Blood Total RNA MiniPrep Kit (Axygen, US). Quantitative PCR was performed in Chromo real-time PCR system (Bio-rad, US) with primers. The sequences of primer pairs used in present study were *LC3B*-forward: AAG GCG CTT ACA GCT CAA TG, *LC3B*-back: CTGGGAGGCATAGACCATGT; *B2M*-forward: AGA TGA GTA TGC CTG CCG TG; *B2M*-back: TCA TCC AAT CCA AAT GCG GC. *LC3B* levels were expressed as ratios relative to *B2M* mRNA in each sample.

### Enrollment of melanoma patients

43 patients (age ≥ 18 years) with histologically confirmed melanoma that would receive surgical removal of the melanoma tissue were solicited sequentially at Peking University Shenzhen Hospital, Shenzhen, Guangdong, China. Primary cutaneous, mucosal melanoma and unknown primary melanoma were eligible; however, primary ocular melanoma was excluded. The Institutional Review Board of Peking University (1120 Lianhua Rd., Futian District, Shenzhen, China) approved the ethic review (Ethical Approval No.: PKUSZH-20180027). Each patient signed the informed consent and knew their remaining melanoma tissue sample in biopsy after surgery will be used in the current study. All methods were performed in accordance with the relevant guidelines and regulations. Detailed patient information was listed in Supplementary Table S1.

### ATP-TCA chemosensitivity assay

An excision biopsy of melanoma lesion was done in every surgery and preprocessed by the Department of Pathology. Remained tissues after biopsies were used in Chemosensitivity testing. It was done using a nonclonogenic ATP-TCA assay (DCS Innovative Diagnostic Systems, Hamburg, Germany). According to the instruction, tissue samples were minced and enzymatically dissociated. After depleted of red blood cell and debris, cell viability was assessed by trypan blue dye exclusion (no less than 25%). Cell suspension (2 × 10^4^ per well, 96 well plate) was treated with Sal at five different dilutions (0.0625, 0.125, 0.25, 0.5 and 1 μM) for 7 days, each tested in triplicates. Cells ATP content was quantified by a luciferin-luciferase luminescence reaction using microplate luminometer (Berthold Detection Systems, Pforzheim, Germany)^49^. Cells incubated without Sal were used as reference for 100% tumor cell viability. IC_50_ of Sal were calculated in every sample. Here we used inhibition rate of Sal (1 μM, 7 days’ treatment) on melanoma cells in the regression analysis for it was more comprehensible here.

### Statistical analysis

All statistical analyses were performed using GraphPad Prism 7 (software, La Jolla, CA) and SPSS version 17.0 software (SPSS, Inc., Chicago, IL). The experimental data were presented as mean ± SD from at least three independent experiments and were assessed by Student’s t-test or one-way ANOVA. *P* values of < 0.05 (*), < 0.01(**), and < 0.001 (***) were considered significant. A linear regression analysis was used to estimate the correlation between sensitivity of Sal and expression of *LC3B* mRNA.

## Supplementary information

Supplementary Information 1.
