# Supplementary material for: Salinomycin induces autophagic cell death in salinomycin-sensitive melanoma cells through inhibition of autophagic flux
Source: Sci Rep. 2020 Oct 28;10:18515. doi: 10.1038/s41598-020-75598-1 (PMC7595060; doi:10.1038/s41598-020-75598-1)

# Salinomycin Induces Autophagic Cell Death in Salinomycin-Sensitive Melanoma Cells through Inhibition of Autophagic Flux

**Yajing Liu[1,4#], Yinghua Hao[1,#], Yuxia Li[1], Yadan Zheng[1], Jiajing Dai[1], Fubo Zhong[1], Wei Wei[2*], Zhengyu Fang[1,3*]**

[1]Biomedical Research Institute, Shenzhen Peking University-Hong Kong University of Science and Technology Medical Center, Shenzhen, 518036, China;

[2] Department of Thyroid and Breast Surgery , Peking University Shenzhen Hospital, Shenzhen, 518036, China;

[3]Clinical Research Institute, Shenzhen Peking University-Hong Kong University of Science and Technology Medical Center, Shenzhen, 518036, China;

[4]Department of Medical Genetics, Institute of Basic Medical Sciences, Chinese Academy of Medical Sciences, Beijing, 100005, China.

[#]These authors contributed equally to this work.

*Supplementary Data*

**Supplementary Fig.S1**

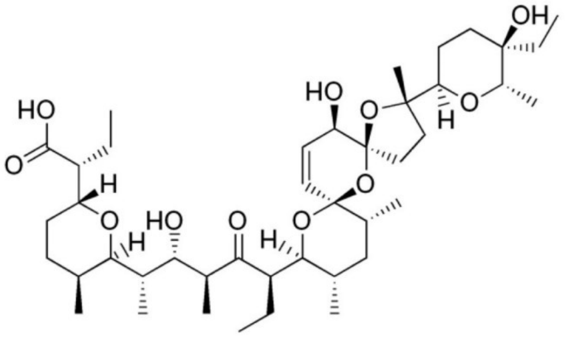

**Supplementary Fig.S1: The structural formula of salinomycin.**

**Supplementary Fig.S2**

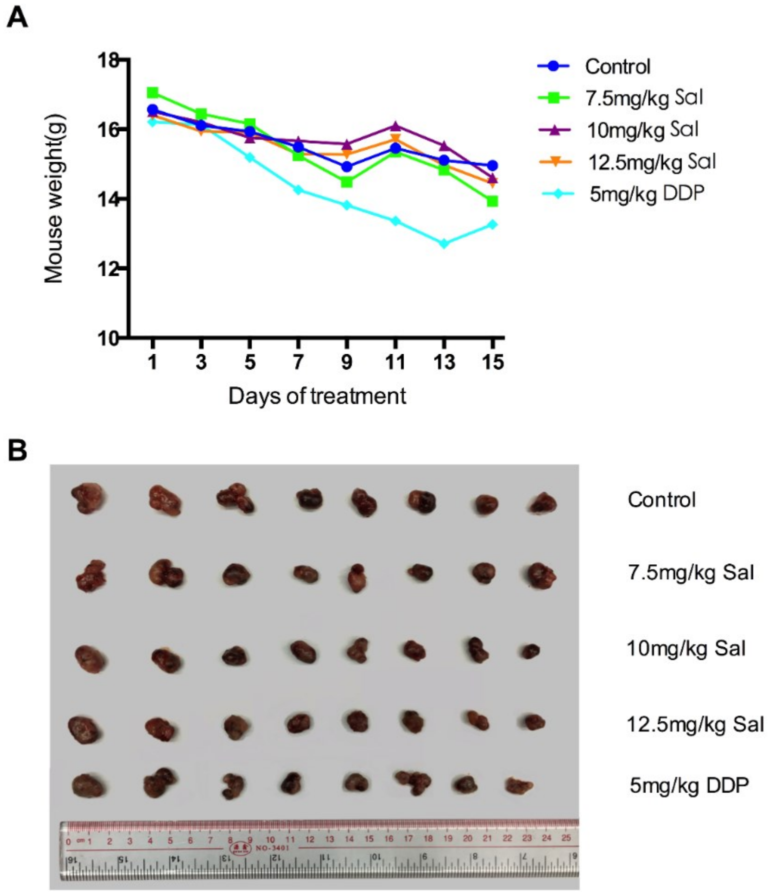


**Supplementary Fig.S2: Sal had an inhibitory effect on the tumor growth *in vivo*.** (**A**) The body weight curve of five groups during 15 days of treatment. (**B**) The photo of mice tumor tissues dissected on the 15th day of treatment.

**Supplementary Fig.S3**

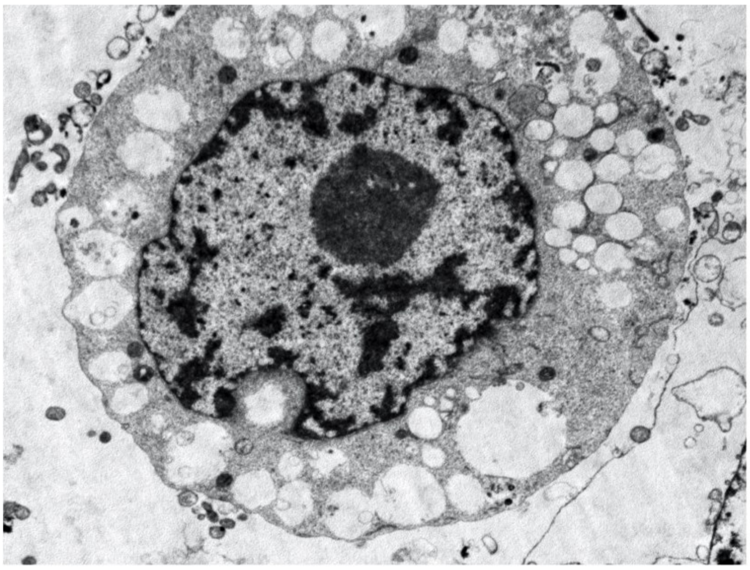

**Supplementary Fig.S3: Typical apoptosis morphological features were observed in a few SK-Mel-19 cells.** EM image showing chromatin condensation and margination in 1.0μM Sal-treated SK-Mel-19 cell.

**Supplementary Fig.S4**

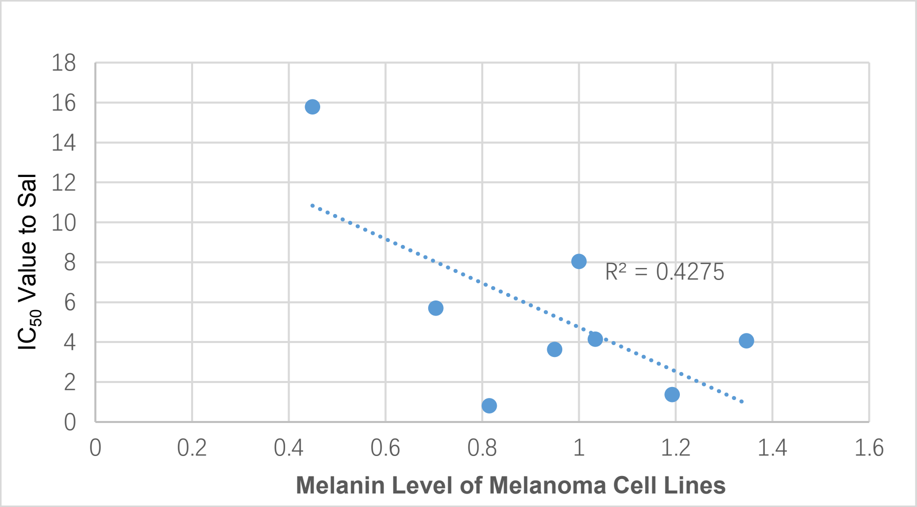


**Supplementary Fig.S4: There is no correlation between the sensitivity to Sal and the pigmentation of eight melanoma cell lines.**

**Supplementary Table S1: Detailed information of clinical characteristics of melanoma patients**

| No. | Gender | Age | Location | Stage | Smoking history | Family history |
|---|---|---|---|---|---|---|
| 1 | male | 51 | foot | II | yes | none |
| 2 | male | 63 | neck | I | yes | none |

| | | | | | | |
|---|---|---|---|---|---|---|
| 3 | male | 35 | face | I | yes | yes |
| 4 | female | 59 | foot | III | none | none |
| 5 | male | 62 | hand | II | yes | none |
| 6 | female | 52 | arm | II | none | none |
| 7 | male | 45 | back | III | none | yes |
| 8 | male | 66 | foot | IV | yes | none |
| 9 | male | 39 | body | II | yes | none |
| 10 | male | 54 | arm | II | yes | none |
| 11 | female | 36 | face | II | none | none |
| 12 | female | 38 | foot | III | none | none |
| 13 | male | 62 | leg | IV | yes | none |
| 14 | male | 60 | face | I | yes | none |
| 15 | female | 43 | chest | II | none | none |
| 16 | male | 50 | back | III | none | none |
| 17 | male | 33 | hand | II | yes | none |
| 18 | female | 31 | abdomen | II | none | none |
| 19 | male | 58 | neck | I | none | none |
| 20 | male | 53 | hand | II | yes | none |
| 21 | male | 57 | foot | IV | yes | none |
| 22 | male | 47 | finger | I | yes | none |
| 23 | female | 40 | foot | II | none | none |
| 24 | female | 33 | scalp | III | none | yes |
| 25 | female | 67 | leg | IV | none | none |
| 26 | male | 53 | abdomen | II | yes | none |
| 27 | male | 56 | foot | III | none | none |
| 28 | female | 63 | scalp | II | none | none |
| 29 | male | 40 | arm | II | yes | none |
| 30 | male | 47 | foot | III | yes | none |
| 31 | male | 57 | leg | IV | yes | none |
| 32 | female | 50 | foot | II | none | none |
| 33 | male | 55 | chest | II | none | none |
| 34 | female | 28 | leg | III | none | yes |
| 35 | male | 26 | hand | I | none | none |
| 36 | male | 72 | finger | II | yes | none |
| 37 | female | 41 | face | II | none | none |
| 38 | male | 60 | neck | II | yes | none |
| 39 | female | 37 | back | III | none | none |
| 40 | male | 46 | foot | IV | none | yes |
| 41 | male | 61 | foot | II | yes | none |
| 42 | male | 51 | chest | III | yes | none |
| 43 | male | 48 | foot | II | none | none |

**Supplementary Table S2: Some cell lines that were sensitive to Sal were not sensitive to cisplatin.**

Comparison of $IC_{50}$ of Sal and DDP in eight melanoma cell lines.

| Melanoma cell lines | $IC_{50}$ of Sal | $IC_{50}$ of DDP |
|---|---|---|
| M7 | 8.05 | 1.57 |
| M8 | 15.78 | 8.88 |
| M21 | 1.38 | 1.68 |
| M29 | 3.63 | 8.91 |
| SK-MEL-1 | 4.15 | 5.37 |
| SK-MEL-19 | 0.82 | 10.16 |
| SK-MEL-103 | 4.07 | 6.95 |
| A375 | 5.71 | 11.43 |

**Uncropped Western Blot Images**

Figure 2B-Cleaved PARP1

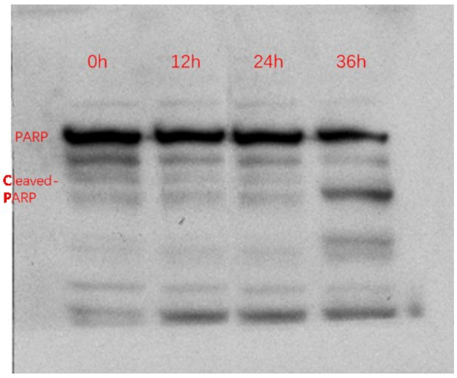


Figure 2B-Cleaved Caspase3

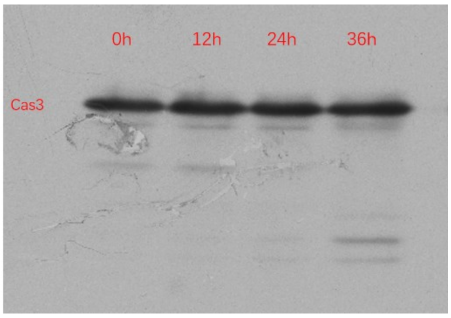


Figure 2B-Caspase9&Cleaved Caspase9&B2M

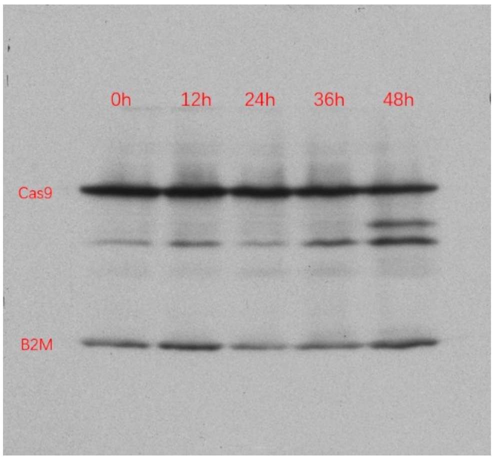

Figure 2C-Cleaved Caspase3

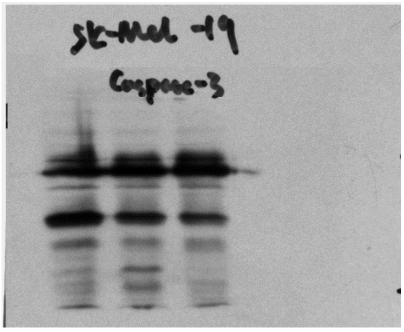


Figure 2C-B2M

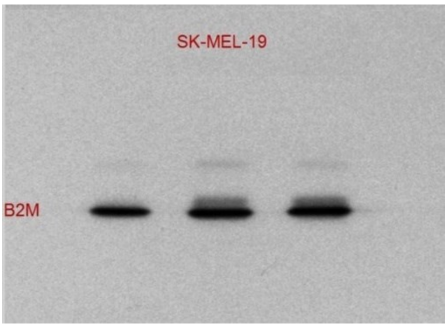


Figure 3A-LC3

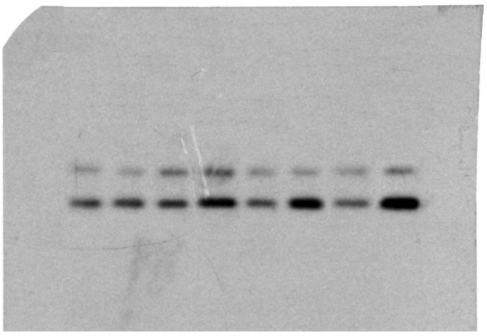

Figure 3A-B2M

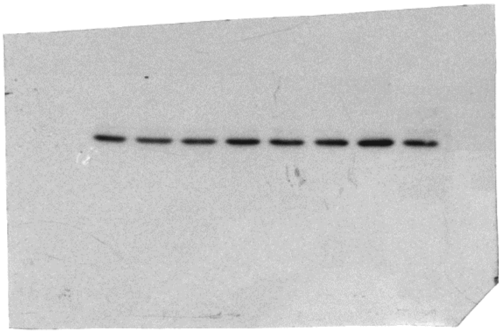

Figure 3B-Beclin-1

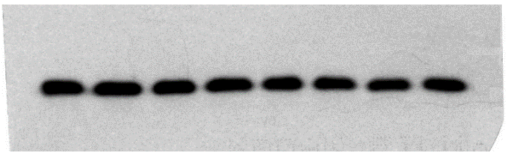

Figure 3B-B2M

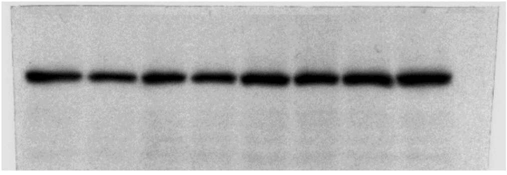

Figure 4A-P62&B2M

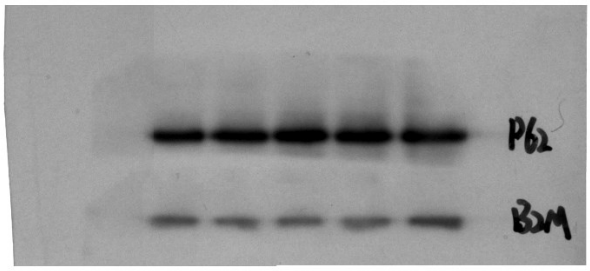


Figure 4D-Ubiquitin

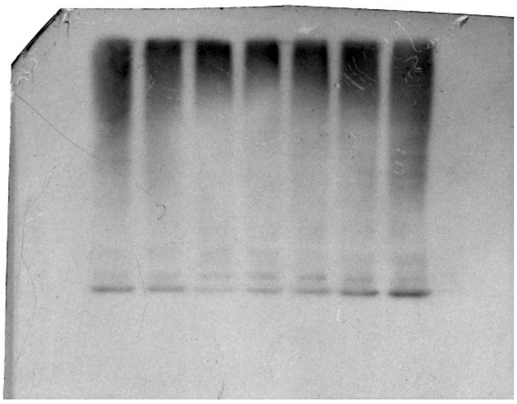

Figure 4D-B2M

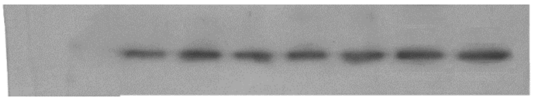

Figure 4E-LC3&B2M

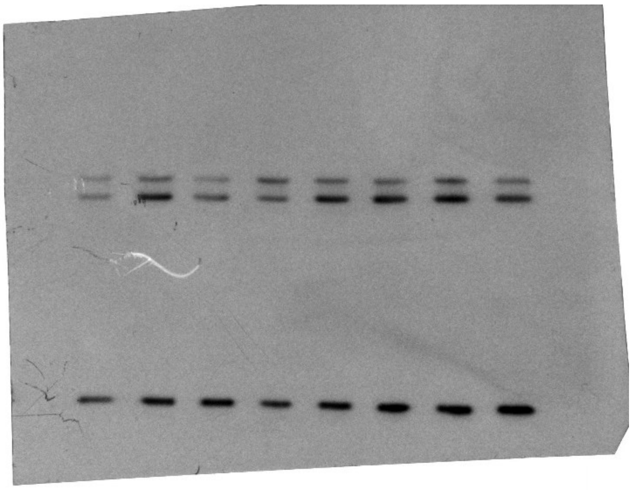

Figure 6B-Bip/GRP78

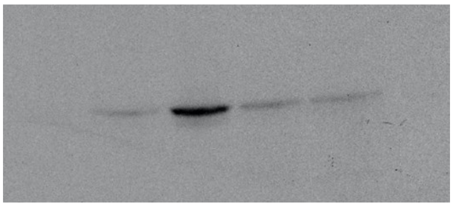

Figure 6B-IRE1a

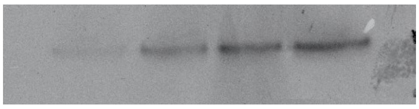

Figure 6B-P-PERK

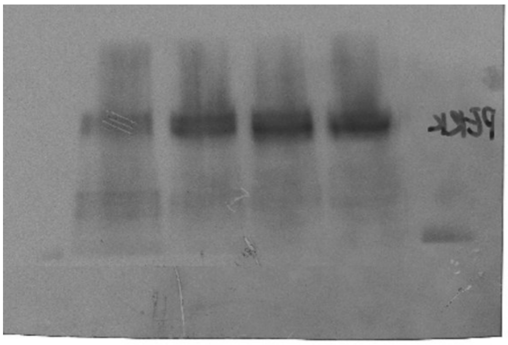

Figure 6B- P-eIF2a

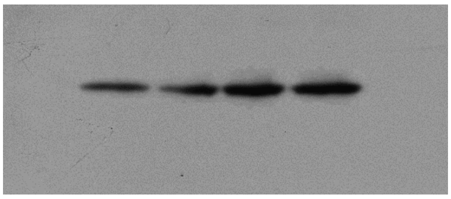

Figure 6B- CHOP &B2M

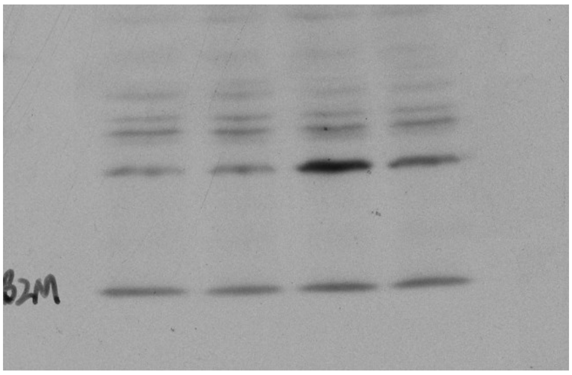

Supplement: Supplementary file 1 — Supplementary Information 1. [file 41598_2020_75598_MOESM1_ESM.pdf]
